# Low Concentration of Sodium Butyrate from Ultrabraid+NaBu suture, Promotes Angiogenesis and Tissue Remodelling in Tendon-bones Injury

**DOI:** 10.1038/srep34649

**Published:** 2016-10-03

**Authors:** Donghui Liu, Silvia Passos Andrade, Pollyana Ribeiro Castro, John Treacy, Jason Ashworth, Mark Slevin

**Affiliations:** 1 School of Healthcare Science, GMBC, Manchester Metropolitan University, Manchester, United Kingdom; 2 Department of Physiology and Biophysics, Institute of Biological Sciences, Federal University of Minas Gerais, Brazil; 3 Smith & Nephew Research Centre, York Science, Park Heslington, York, UK; 4 University of Medicine and Pharmacy, Tirgu Mures, Romania

## Abstract

Sodium butyrate (NaBu), a form of short-chain fatty acid (SCFA), acts classically as a potent anti-angiogenic agent in tumour angiogenesis models, some authors demonstrated that low concentrations of NaBu may contribute to healing of tendon-bone injury in part at least through promotion of tissue remodelling. Here, we investigated the effects of low-range concentrations of NaBu using *in vitro* and *in vivo* assays using angiogenesis as the primary outcome measure and the mechanisms through which it acts. We demonstrated that NaBu, alone or perfused from the UltraBraid+NaBu suture was pro-angiogenic at very low-range doses promoting migration, tube formation and cell invasion in bovine aortic endothelial cells (BAECs). Furthermore, cell exposure to low NaBu concentrations increased expression of proteins involved in angiogenic cell signalling, including p-PKCβ1, p-FAK, p-ERK1/2, p-NFκβ, p-PLCγ1 and p-VEGFR2. In addition, inhibitors of both VEGFR2 and PKCβ1 blocked the angiogenic response. In *in vivo* assays, low concentrations of NaBu induced neovascularization in sponge implants in mice, evidenced by increased numbers of vessels and haemoglobin content in these implants. The findings in this study indicate that low concentrations of NaBu could be an important compound to stimulate angiogenesis at a site where vasculature is deficient and healing is compromised.

Sodium butyrate (NaBu), a form of short-chain fatty acid (SCFA), has been reported to exert profoundly effects on mammalian cells *in vitro* and modify the activity of many types of cells^1^,^2^, such as colorectal cancer cells^3^,^4^,^5^. It has been shown to inhibit angiogenesis at high concentrations. It is recognized to serve as a major energy substrate for colonocytes, exert potent effects on epithelial cells, induce cell replication and proliferation, act as an inducer or inhibitor of cell differentiation, induce apoptosis^6^,^7^,^8^,^9^, and lead to cell growth arrest or cell death^10^,^11^. It can also modify cell morphology, possibly through its effects on the cytoskeleton^1^,^2^,^12^. Many biological properties of NaBu are well documented; for example, it can inhibit oral cancer cell growth by arresting the cell cycle in G1 phase^13^, whilst, others have demonstrated that NaBu exerts a strong permissive effect on hepatocyte *in vitro*^14^. Normal tissue physiological concentrations of sodium butyrate are between 0.1–0.5 ug/cm^3^ far below the normal experimental range used in cell culture studies (Meijer *et al*.^15^).

Significant work has reported that high concentrations NaBu may inhibit histone deacetylase, which results in hyperacetylation of proteins inhibiting DNA replication^2^,^16^. NaBu promotes hyperphosphorylation of the nuclear proteins HMG 14 and HMG 17^17^,^18^ which are associated with active chromatin. Thus, it is believed that the nucleus is the essential cell compartment which NaBu acts upon^19^,^20^, while, the effects of NaBu on gene/protein expression in the non-epithelial cell populations, including the microvascular endothelial cells, are presently unknown.

To our knowledge, there are currently no reports on the biological functions of low- range concentrations of NaBu within vascular cells.

Recently, some reports have described the effects of NaBu on the tendon-bones injury repair and remodelling^21^,^22^. It was shown that UltraBraid suture typically used in the therapy of tendon-bones injury, when impregnated with NaBu enhanced vascular repair and remodelling in a rabbit model (now undergoing clinical trials), suggesting NaBu may promote local tissue remodelling in tendon-bones injury improving strength and healing.

The Ultrabraid+NaBu suture promotes tendon-bones injury repair and remodelling, with evidence of increased angiogenesis, a short healing time and better clinical outcomes^22^. Hence, here, we aimed to investigate the mechanisms through which NaBu enhances tendon-bones injury via modulation of angiogenesis.

In relation to this, moderate levels of NaBu was shown to down-regulate the expression of vascular endothelial growth factor (VEGF) by affecting the activation of HIF-1α, a key regulator of VEGF on Caco-2 cells, by down-regulating the expression of nuclear HIF-1α^23^,^24^,^25^,^26^, HIF-1α expression can be regulated through several post-transcriptional mechanisms. In addition, NaBu has been shown to modulate the activity of nuclear transcription factor-kappa beta (NF-κβ) in a number of different cell types, including colon cancer cells^23^. Hence, the anti-proliferative effect of NaBu on transformed cells is well established, and NaBu has emerged as an antiangiogenic agent that directly represses the expression of angiogenic ligands or indirectly interferes with endothelial cell proliferation/enzymes required for angiogenesis^27^,^28^,^29^.

In this study, the mechanisms through which NaBu modulates angiogenesis at low concentrations have been examined and their possible relationship to enhance repaired of tendon-bone injury via the Ultrabraid + NaBu suture discussed.

## Materials and Methods

### Materials

Collagen and gelatine were purchased from Sigma Aldrich UK. The Sutures and NaBu were kindly provided by Smith & Nephew Inc, USA. Matrigel^TM^ gel was purchased from BD Biosciences International, UK. Foetal bovine serums (FBS), trypsin, phosphate buffered saline (PBS) and Dulbecco’s Modified Eagle’s Medium (DMEM) were purchased from Lonza, UK.

#### Cell culture

Bovine aortic endothelial cells (BAECs) were prepared as described by Sattar *et al*.^30^. BAECs were cultured in DMEM supplemented with 10% FBS, 2 mM glutamine and 1% antibiotics (100 U/ml penicillin and 100 μg/ml streptomycin) in T25 flask pre-coated with 0.1% gelatin and incubated in a water-saturated, 5% CO_2_ incubator at 37 °C. For each experiment, BAECs were serum starved (incubated with DMEM supplied with 1% FBS) for 24 hours prior to the assay. Generally, about 80% confluent BAECs incubated in serum poor medium (SPM, contains 1% FBS) overnight and up to 24 hours. NaBu at low-range concentrations (1 ng/ml, 5 ng/ml, 10 ng/ml, 25 ng/ml, 50 ng/ml, 0.1 μg/ml, 1.0 μg/ml, 5.0 μg/ml, 10.0 μg/ml, 25 μg/ml, 50.0 μg/ml and 100.0 μg/ml) were investigated in the study. All experiments were repeated three times and samples were prepared in triplicate (n = 9) in all cases. The BAECs were used between passages 5–10 for all the experiments.

### *In vitro* studies

#### Endothelial cell tube formation assay

BAEC were cultured in complete growth medium (DMEM with 10% FBS) in a water-saturated incubator at 37 °C and 5% CO_2_. When cells reached at about 80% confluence, the growth medium was changed to SPM containing only 1% FBS and incubated for a further 24 hours. Cells were trypsinised (10% trypsin-EDTA solution) and re-suspended at 1 × 10^6^ cells/ml. Cells were mixed thoroughly with an equal volume of liquified Matrigel^TM^ gel, together with NaBu at variety of low-range concentrations (as described previously). The cell/Matrigel^TM^ gel mixture was put into 48-well plate and left for 1 hour to allow the Matrigel^TM^ gel to polymerize at 37 °C. The NaBu-impregnated Matrigel^TM^ gel/cell samples were incubated in SPM for 24 hours. Fibroblast growth factor-2 (FGF-2) at concentration of 25 ng/ml was used as positive control in place of NaBu. After 24 hours incubation, cells were fixed with 50 μl/well of 4% paraformaldehyde (PFA) for 15 minutes at room temperature (RT). Tube formation in each well was assessed by phase contrast microscopy and images were taken using a digital camera (Zeiss).

### Endothelial cell migration assay

BAECs were cultured on 1 mm × 1 mm glass cover slips in complete growth media and incubated in a water-saturated incubator at 37 °C and 5% CO_2_. When cells reached about 80% confluence, the growth medium was replaced with SPM and incubated for further 24 hours. Adherent cells were then scratched in one single continuous line across the glass cover slip using a razor blade and then washed twice with warm PBS. Cells were then incubated in SPM containing NaBu at a various of low-range concentrations (as described previously) and incubated for further 24 hours. Again, FGF-2 (at 25 ng/ml) was used as positive control. Cell migration was assessed by phase contrast microscopy and images were taken using a digital camera (Zeiss). Both migration distance and number of migrated cells were measured.

### Endothelial cell invasion assay

BAECs migration and tube formation were also enhanced by NaBu in the sandwich Matrigel^TM^ gel, suggesting a neoangiogenic effect at low-range concentrations of NaBu. NaBu-induced ECs tube formation and invasion assay was performed using two layers of sandwiched Matrigel^TM^ gel. BAECs were mixed with Matrigel^TM^ gel as previously described and cultured for 24 hours at 37 °C and 5% CO_2_ to enable tube-like structures to form. A second layer of Matrigel^TM^ gel, containing various low-range concentrations (as described previously) of NaBu, was applied in direct contact with the first layer of Matrigel^TM^ gel. After further 24 hours incubation, cells were fixed with 50 μl/well of 4% PFA for 15 minutes at RT. ECs tube formation and invasion were assessed by using phase contrast microscopy and images were captured using a digital camera (Zeiss).

### Ultrabraid+NaBu suture induced endothelial cell tube formation assay

BAEC were cultured in complete growth medium in a water-saturated incubator at 5% CO_2_ and 37 °C. When cells reached about 80% confluence, the growth medium was replaced with SPM and incubated for a further 24 hours. Cells were trypinised and re-suspended at 2 × 10^6^ cells/ml in complete media before adding 4 × 10^5^ cells onto pre-polymerized Matrigel^TM^ gel in a 24-well plate. The plate was then incubated for 4 hours at 5% CO_2_ and 37 °C to enable cells to attach to the Matrigel^TM^ gel. After checking for adhesion to the first layer of Matrigel^TM^ gel, the media was aspirated from each well and a NaBu-impregnated ultrabraid suture was applied to the middle of each well. Immediately a second layer of Matrigel^TM^ gel was added onto the first layer of the Matrigel^TM^ gel to form a Matrigel^TM^ gel sandwich and allowed to polymerize for 1 hour at 37 °C. The Matrigel^TM^ gel sandwich was incubated for 24 hours at 37 °C and 5% CO_2_ following the addition of 500 μl low serum (1% FBS) growth medium per well. A general surgical suture was used as described above as a control in place of the impregnated ultrabraid suture.

### Measurement of suture release kinetics of sodium butyrate

6-well plates were used to incorporate various lengths of suture within a Matrigel^TM^ gel tube-formation protocol system and with and without BAECs. Following insertion and incubation of the plates at 37 °C and 5% CO_2_, 3 mm Strips of Matrigel^TM^ gel were accurately cut on each side of the suture using a scalpel at time zero, 4 h and 24 h (n = 5 per test). Control liquefied Matrigel^TM^ gel +/− butyrate concentration range (spiked) was used to construct a standard curve.

The matrix was liquefied with 1.5 U/ml dispase I and the reaction stopped using 5 mM EDTA. The Matrigel^TM^ gel + dispase I - was spiked with different concentrations of butyrate 0.01 micrograms-100 micrograms-pilot tested and quantified by a standard HPLC protocol.

### Kinexus phopho-protein microarray analysis

BAECs were seeded in completed medium in a 6-well plate at a cell concentration of 2.5 × 10^5^ cells/ml/well. After 48 hours incubation, the growth medium was replaced with SPM and incubated a further 24 hours. The following day, 1 μg/ml of NaBu was added to each well and allowed to incubation at 37 °C and 5% CO_2_ for 10 minutes and 1 hour respectively. To inspect the variation of the signalling protein expression induced by the NaBu, the phosphor-protein microarray of 880 phopho-site proteins, Kinexus^TM^ Antibody Microarray (KAM)-1.2 was performed by Kinexus Bioinformatics (Vancouver, Canada).

Proteins from NaBu-stimulated and un-stimulated cells were extracted according to Kinexus service protocols. Briefly, cells were washed twice with cold PBS, then protein was extracted using a kinexus cell lysis buffer which was previously described^31^. Cell extracts were then collected and sonicated three times for 15 seconds, and protein concentrations were measured by the Bradford protein assay using a spectrophotometer and according to the manufacturer’s instructions (Bio-rad, Munchen, Germany). For each sample, protein (200 μg in 100 μl) was transferred to a fresh 1.5 ml Eppendorf screw cap and then was sent to Kinexus for analysis.

### Western blot Analysis

Whole cell lysates were solubilized in a 2-fold concentrated sample buffer, and 30 μg of total protein sample were separated by SDS-PAGE. Proteins were transferred to a nitrocellulose membrane (Whatman, protran BA85, Germany), probed with appropriate primary antibodies (Abcam) as indicated and incubated over-night at 4 °C. Membranes were then incubated with an HRP IgG secondary antibody. Bound HRP IgG was visualized using enhanced chemi-luminescence (ECL, Thermo scientific, UK) with Western Blotting Substrate. Excess liquid was absorbed and the membrane exposed to ChemiDoc Touch Imaging System (Bio-rad, UK). Band intensity was quantified with Image-Lab software (supplied by ChemiDoc Touch Image System, Bio-rad, UK). The fold increase of each specific protein was determined by the ratio of band intensities of treated and untreated cells, normalized to loading control (α-tubulin, Abcam).

BAECs were seeded in complete medium in a 6-well plate at a cell concentration of 2.5 × 10^5^/ml/well. After 48 hours incubation, the medium was replaced with SPM and cells incubated for a further 24 hours. Next, 1 μg/ml NaBu was added and the cells incubated for 10 min, 20 min, 30 min and 1 hour at 37 °C. In addition, BAECs cultured under the same conditions were exposed to PKC inhibitor (Ruboxistaurin, optimised and used at 0.2 nM-see Kunt *et al*. for information of the high specificity of this inhibitor)^32^ for 4 hours and 24 hours respectively prior to addition of NaBu (1 μg/ml) in some experiments in order to identify any potential blocking effects on PKC signalling. In addition, m-CRP protein was applied to the assay (as an alternative positive control expected to show a lack of inhibition with the PKC inhibitor since its signalling does not pass through this pathway). Briefly, the starved BAECs were incubated +/− PKC inhibitor (Ruboxistaurin, 0.2 nM) for 4 hours, then 5 μg/ml of m-CRP protein was introduced to the cells for 10 minutes, untreated cells were as control, and m-CRP treated cells (without inhibitor) were used as a positive control. The same method was used as for the VEGFR inhibition assay which was as follows: BAECs were pre-incubated with VEGFR receptor blocker (1:100 dilution) for 4 hours, then the cells were treated with NaBu (1 μg/ml) for 20 min. FGF-2 (25 ng/ml) was as positive control. After rapid washing with ice-cold PBS, cells were lysed with 200 μl/well of ice-cold radioimmunoprecipitation (RIPA) buffer (pH 7.5) containing 25 mM Tris-HCl, 150 mM NaCl, 0.5% sodium deoxycholate, 0.5% SDS, 1 mM EDTA, 1 mM sodium orthovanadate (EGTA), 1 mM phenylmethylsulfonyl fluoride (PMSF), 1% Triton × 100 and 1 μM leupeptin. The protein concentration of cell lysates was determined using the Bradford protein assay (Bio-rad, Munchen, Germany) and equal quantities of proteins (30 μg) were mixed with 2× Laemmli sample buffer, boiled in a water bath for 15 min then centrifuged. Samples were separated along with pre-stained molecular weight markers (32,000–200,000 kDa) by 12% SDS-PAGE. Proteins were electro-transferred (Hoefer, Bucks, UK) onto nitrocellulose filters (1 h) and the filters were blocked for 1 h at room temperature in TBS-Tween (pH 7.4) containing 1% bovine serum albumin (BSA). Filters were stained with the primary antibodies diluted in the blocking buffer, overnight at 4 °C on a rotating shaker. The following primary antibodies (1:1000) from Abcam (Abcam, UK), p-ERK1/2 (S404), p-FAK, p-NFκβ, p-PKCβ1, p-PLCγ1 and p-VEGFR2 were applied. After washing (5 × 10 min in TBS-Tween at room temperature), filters were stained with either goat anti-rabbit or rabbit anti-mouse HRP-conjugated secondary antibodies diluted in TBS-Tween containing 5% de-fatted milk (1:2000, 1 h, room temperature) with continuous mixing. After a further five washes in TBS-Tween, proteins were visualized using enhanced chemi-luminescent detection (ECL, Thermo scientific, UK) and semi-quantitatively identified fold differences compared with house-keeping controls determined using Image-Lab software. All experiments were repeated at least twice and a representative example is shown.

### *In vivo* study

#### Animal Ethics Statement

The use of animals and procedures for this study was approved by the Ethics Committee in Animal Experimentation (CETEA) of Federal University of Minas Gerais (Protocol no. 267/ 2014). All surgery was performed under ketamine and xylazine anesthesia, and all precautions were made to minimize suffering.

#### Animals

A total number of 104, eight-weeks-old male C57/Bl mice from Centro de Bioterismo of the Federal University of Minas Gerais (UFMG) were used. The study design, replicates per test and total animal numbers used are shown in Table 1. Mice were maintained in individual cages with food and water ad libitum, with controlled temperature and humidity in the animal house at the Department of General Pathology, Institute of Biological Sciences. Experiments were carried out in accordance with approved procedures granted by the Ethics Committee in Animal Experimentation of UFMG. All animal care and procedures were approved (protocol number 43/2014) and complied with the guidelines established by the Institutional Committee on Animal Welfare (CETEA) of Federal University of Minas Gerais. Efforts were made to avoid all unnecessary distress to animals.

The light/dark cycle was 12:12 h with lights on at 7:00 am and lights off at 7:00 pm. Post-operatively, animals were monitored for any signs of infection at the operative site.

All tests were carried out in a double-blinded fashion and details of methodology are given below.

### Statistical evaluation

All statistical tests were performed using SPSS. A total of 64 animals were used for the implant experiments (n = 8 per group, total = 64 animals) based on advice from an independent medical statistician together with information gathered from protocol developers in respect of minimum numbers required to gather statistically significant data. This advice took into account possible mortality (n = 0–1 per group) and incorrect implant (n = 0–1 per group), with statistical significance being tolerable at n > 5 per group based on our previous studies^33^. For histology, based on our previous experience a minimum of n = 5/group (total n = 40 animals) was sufficient to give statistical significance in ANOVA tests when comparing treated groups with the control group. Furthermore, based on previous statistical analysis, we have established that counting 5 microscopic fields (8533 μm^2^ per field) per treatment group was sufficient to maintain the standard deviation within a 10% variation^34^.

### Implant Methodology

C57/Bl mice (n = 20 per test) were implanted with sponge disks containing NaBu (0.1–100 μg NABU in 40 μl followed by 2 additional doses at 48 hours and 76 hours) or with NaBu-impregnated Ultrabraid sutures. A sub-cutaneous dorsal pouch was prepared for implant deliveries. After 7 days, implants were assessed for haemoglobin content using the Drabkins method. In addition, homogenised supernatants from implants after 7 days incubation were evaluated for N-acetyl-β-D-glucosaminidase (NAG) determination of mononuclear cells infiltration, VEGF expression and histology.

### Implantation of the sponge discs

Mice were anesthetized (i.p.) with a mixture of xylazine (10 mg/kg) and ketamine hydrochloride (100 mg/kg). Their dorsal hair was shaved and the skin wiped with 70% ethanol. A sponge disk made of polyether polyurethane (dimensions = 8 mm diameter, 4 mm thick, volume 0.2 cm^3^) was then aseptically inserted into a subcutaneous pouch made with curved artery forceps through a 1cm long dorsal mid-line incision (treated groups). After the implantation procedure, the animals received the first dose of sodium butyrate (0.1 μg, 0.5 μg, 1.0 μg 5.0 μg, 10.0 μg, 50.0 μg and 100 μg respectively in 40 μl) or phosphate buffered saline (PBS) as control. Additional doses were given at 48 hours and 96 hours post-implantation (following pilot studies using single, double and multiple doses and consideration of the 24–47 hour butyrate metabolism kinetic to ensure replenishment throughout the experiment and maintenance of concentration levels).

At 7 days post-implantation, the animals were sacrificed and implants removed for assessment of haemoglobin content (vascular index), inflammatory marker (N-acetyl-β-D-glucosaminidase, NAG) and VEGF levels. For histological analysis, a separate group of mice (n = 5 for each group) was used.

### Angiogenesis Quantitation

For histology, the sponge implants from all groups (n = 5 for each group) were fixed in 10% buffered formalin (pH 7.4) and processed for paraffin embedding. Sections 5 μm thick were stained with haematoxylin/eosin (H&E) and processed for light microscopy. To perform morphometric analysis of a number of new blood vessels that had vascularised the sponge, images of cross sections obtained from 5 fields of view were captured for each implant with a planapochromatic objective (40x) in light microscopy (final magnification = 400x). Images were digitized through a JVC TK-1270/JCB microcamera and transferred to an analyzer (software: Image-Pro Plus 4.5, Media Cybernetics, Inc.). For statistical analysis, a countable blood vessel was defined as a tube-like structure with a lumen, whether or not it contained red blood cells.

### Haemoglobin Analysis

The extent of the vascularization of the sponge discs was assessed based on the amount of haemoglobin (Hb) detected in the tissue using the Drabkin method. At 7 days post-implantation, animals in both treated and untreated groups were euthanized and the sponge discs carefully removed. Sponges were dissected from adherent tissue, weighed, homogenized (Tekmar TR-10, OH) in 2 mL of Drabkin reagent (Labtest, São Paulo, Brazil) and centrifuged at 4 °C at 12,000 g for 20 min. The supernatants were then filtered through a 0.22 μm Millipore filter. The Hb concentration of the samples was determined spectrophotometrically by measuring absorbance at 540 nm using a colourimetric plate reader and compared against a standard curve of Hb. The content of Hb in the sponge discs was expressed in micrograms of Hb per milligram of wet tissue.

### VEGF analysis

Implants from control or treated groups were removed at day 7 post-implantation, homogenized in PBS pH 7.4 containing 0.05% Tween-20 (Difco/USA) and centrifuged at 4 °C, 10,000 × g for 30 min. VEGF in the supernatant from each sample was measured using an immunoassay kit (R&D Systems, USA) according to the manufacturer’s protocol. Briefly, dilutions of cell-free supernatants were added in duplicate to wells of the assay plate that was coated with a murine monoclonal antibody against VEGF, followed by the addition of a second horseradish peroxidase-conjugated polyclonal antibody against VEGF. After washing to remove any unbound antibody-enzyme reagent, a substrate solution of tetramethylbenzidine (TMB, Sigma-USA) was added to the wells. The colour development was stopped, after 20 min incubation, with sulphuric acid and the absorbance measured at 450 nm on a spectrophotometer (Thermoplate Tekmar). The VEGF concentration (pg/ml) in each sample well was determined by interpolation from a set of recombinant murine VEGF standards provided in the kit. VEGF concentration from implants was then expressed as pg VEGF per mg wet tissue.

### Measurement of NAG (N-acetyl-β-D-glicosaminidase)

The infiltration of mononuclear cells was quantitated by measuring the levels of the lysosomal enzyme N-acetyl-β-D-glucosaminidase (NAG) present in high levels in activated macrophages. Following the Hb measurements the remaining pellets were weighed, homogenized in sodium chloride (NaCl) solution (0.9% w/v) containing 0.1% v/v Triton X-100 (Promega; USA) and centrifuged (3,000 × g; 10 min at 4 °C). Supernatants were incubated for 30 min with an equal volume of 2.24 mM p-nitrophenyl-N-acetyl-beta-D-glucosaminide (Sigma; USA) prepared in citrate/sodium phosphate buffer (0.1 M citric acid, 0.1 M Na_2_HPO_4_; pH4.5). The reaction was stopped by the addition of glycine buffer (0.8 M glycine, 0.8 M NaCl, 0.8 M NaOH; pH 10.6). Hydrolysis of the substrate was determined by measuring the absorbance at 400 nm. NAG activity was expressed as nmol of p-nitrophenol per mg wet tissue.

## Results

### Nanogram concentrations of sodium butyrate induced angiogenesis in vascular endothelial cells

**Figure 1:**
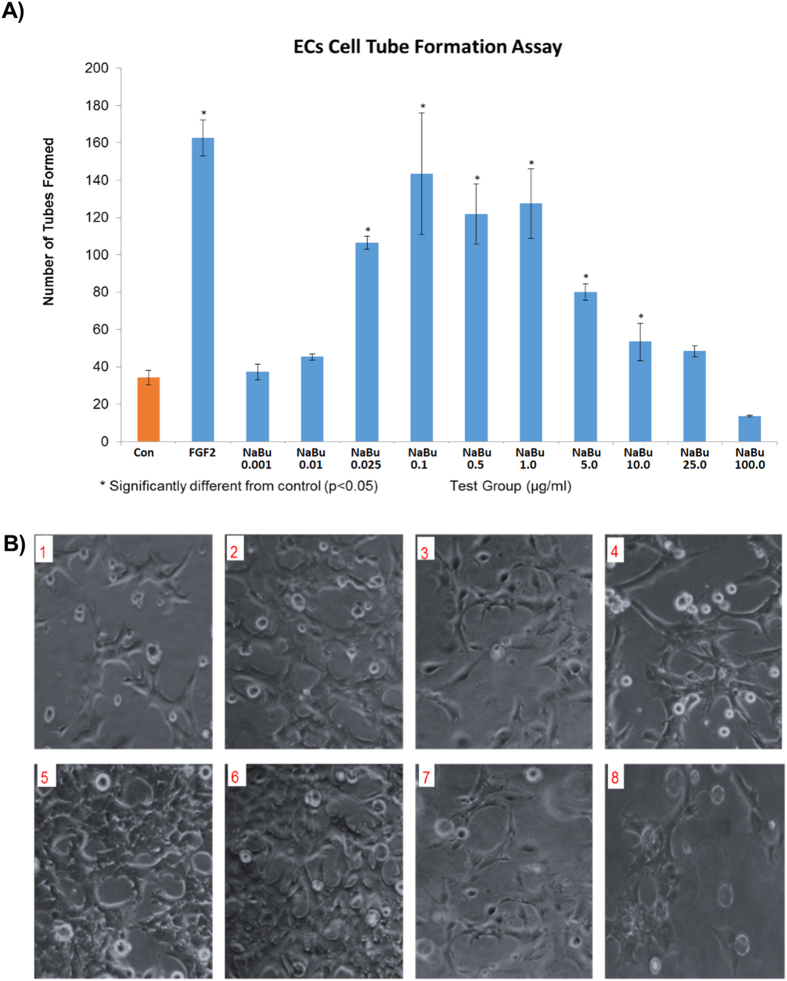
Low dose NaBu induced BAEC tube formation. The bar graph (**A**) indicates that NaBu induced BAEC cells tube formation start from 25 ng/ml, with maximal effects at concentrations of 0.1–1.0 μg/ml Higher concentrations had less effect, and those of 10.0, 25.0, 100.0 μg/ml, showed no significant differences compared to the control. The images (**B**) shows that there were significantly more tube-liked structures when NaBu concentrations were between 0.1 μg/ml (B5) and 1 μg/ml (B6). B1: Control, B2: FGF-2, B3: NaBu 1 ng/ml, B4: NaBu 25 ng/ml, B5: NaBu 0.1 μg/ml, B6: NaBu 1 μg/ml, B7: NaBu 25 μg/ml, B8: NaBu 100 μg/ml. All experiment were repeated at least twice and representative examples are shown.

**Figure 2:**
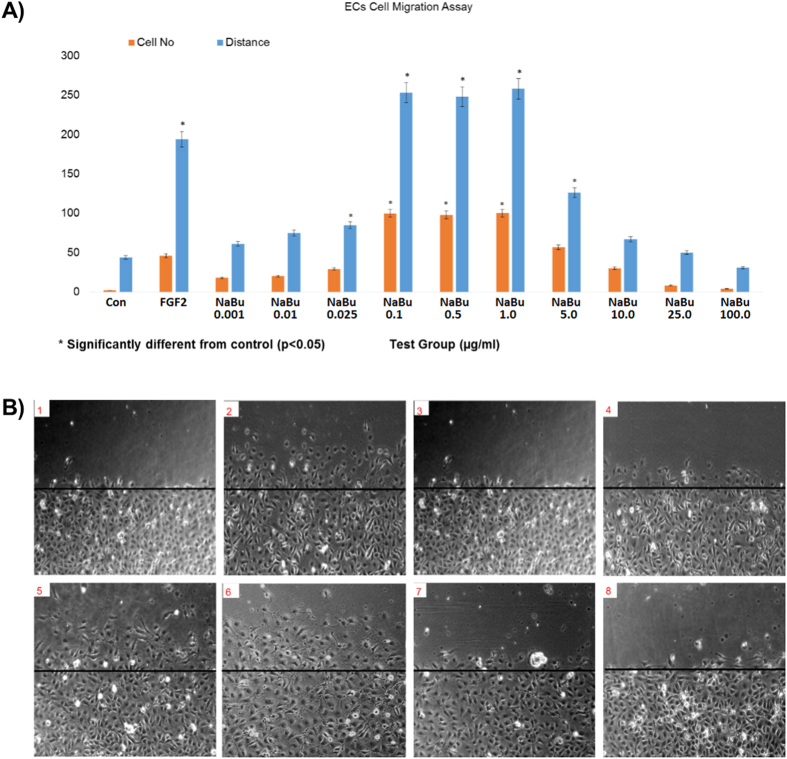
Sodium butyrate induced BAEC cell migration. BAECs treated with NaBu at very low concentrations stimulated BAEC cell migration. FGF-2 at 25 ng/ml was the positive control. The bar chart (**A**) shows that NaBu induced BAEC cells migration substantially at concentrations of 0.1 μg/ml–1.0 μg/ml but there were no significance with concentrations of 5.0 μg/ml and above. The representative images (**B**) show that there were more migrated cells at NaBu concentrations of 0.1 μg/ml (B5) and 1 μg/ml (B6). B1: Control, B2: FGF-2, B3: NaBu 1 ng/ml, B4: 2% NaBu 25 ng/ml, B5: NaBu 0.1 μg/ml, B6: NaBu 1 μg/ml, B7: NaBu 25 μg/ml, B8: NaBu 100 μg/ml. All experiment were repeated at least twice and representative examples are shown.

**Figure 3:**
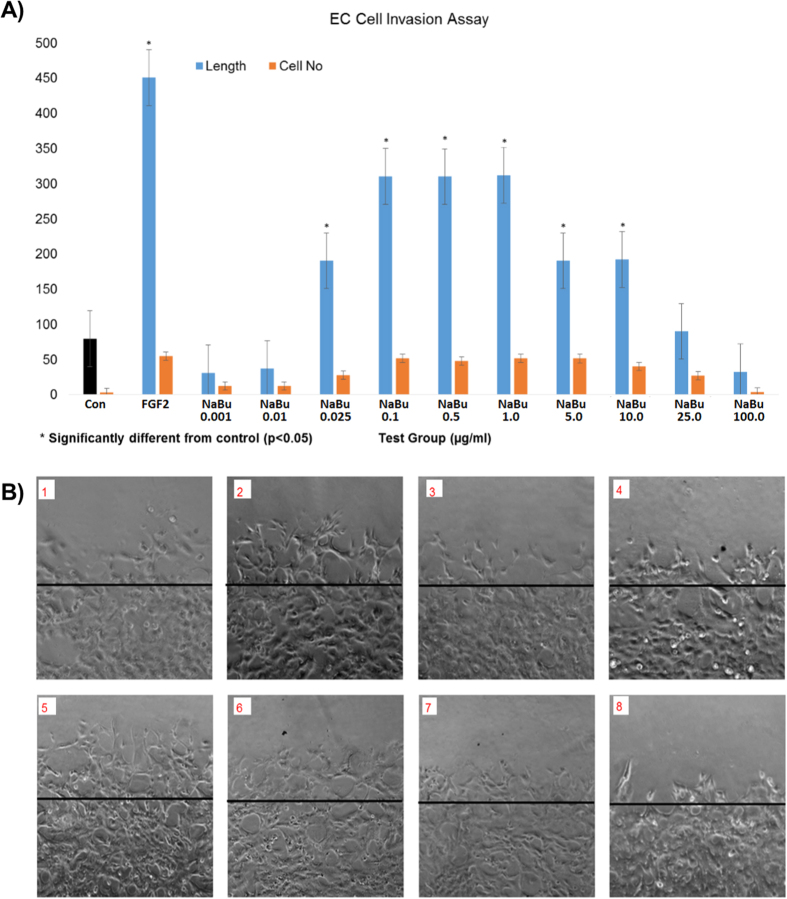
Sodium butyrate induced BAEC cell invasion. Sodium butyrate was introduced to the second layer of growth factor reduced Matrigel at various concentrations and incubated in 2% FBS DMEM medium at 37 °C for 24 hours, the cells without NaBu were used as negative control and FGF-2 at 25 ng/ml was a positive control. The bar chart (**A**) shows that NaBu induced BAEC cells to invade into the second layer of Matrigel, and the cell invasion effects were seen at concentrations 25 ng/ml–1.0 μg/ml, (**B**) The representative images show tube-like structures in a second layer of matrigel at 0.1 μg/ml (B5) and 1 μg/ml (B6). B1: Control, B2: FGF-2, B3: NaBu 1 ng/ml, B4: 2% NaBu 25 ng/ml, B5: NaBu 0.1 μg/ml, B6: NaBu 1 μg/ml, B7: NaBu 25 μg/ml, B8: NaBu 100 μg/ml. All experiment were repeated at least twice and representative examples are shown.

*In vitro* testing showed that addition of sodium butyrate between 25 ng/ml and 1ug/ml to BAECs in Matrigel^TM^ gel caused a significant increase in tube-like-structure formation (Fig. 1). Similar effects were seen in wound recovery using a scratch wound method, where sodium butyrate was effective at 100 ng/ml to 1 ug/ml (Fig. 2). A cell invasion assay was performed also in Matrigel^TM^ gel, where a second outside layer containing sodium butyrate was placed around the already formed tube-like structures. Sodium butyrate from 25 ng/ml to 10 ug/ml was able to significantly increase the invasion of cells and tubes into this second layer over a further 24 h period (Fig. 3).

### Ultrabraid+NaBu suture stimulated tube-like-structure formation of BAECs in matrigel

**Figure 4:**
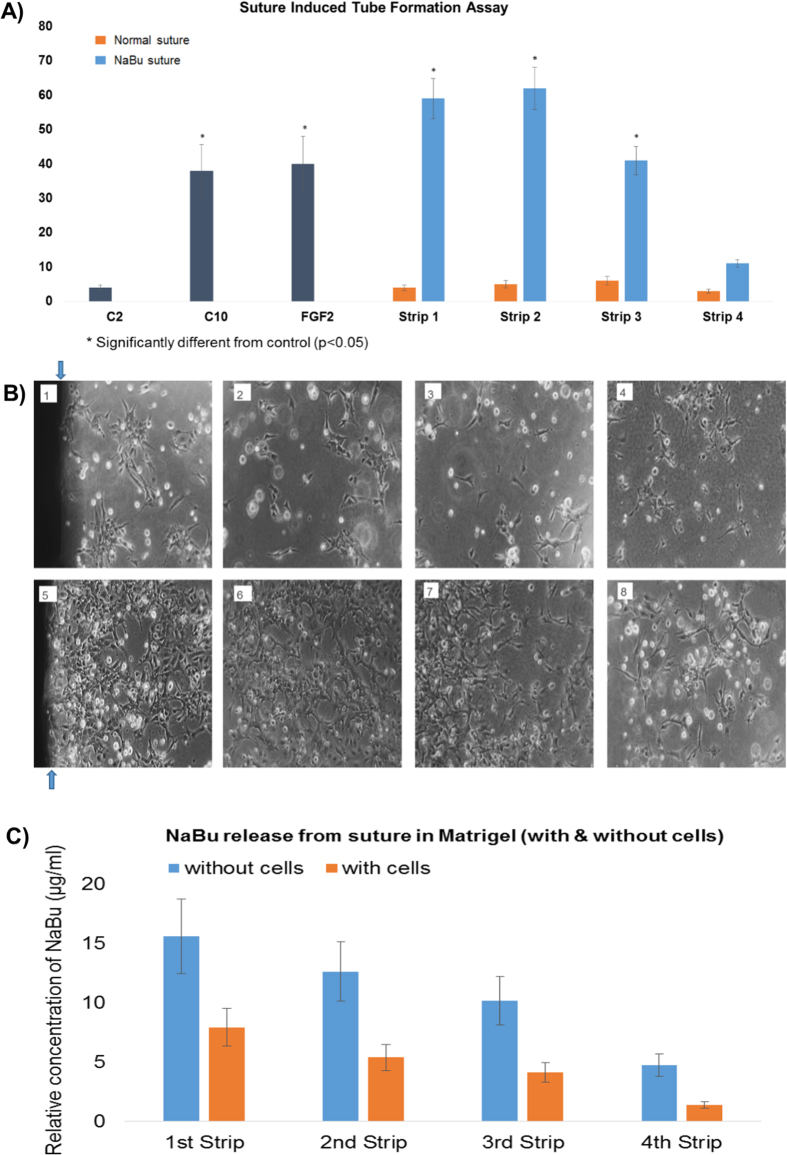
Ultrabraid (NaBu-infused) suture induced tube formation. Sodium butyrate Ultrabraid suture was introduced to the sandwich Matrigel, and the cells were seeded at 1,000,000/ml and incubated in 2% FBS DMEM medium at 37 °C for 24 hours. BAEC cells with general (none-NaBu containing) suture, and cells without suture were used as negative control and FGF-2 at 25 ng/ml was a positive control, and cells incubated in 10% FBS DMEM medium were also used as a secondary positive control. The bar chart (**A**) shows Ultrabraid suture significantly induced BAEC tube formation (blue bar), compared to the general suture (orange bar). (**B**) Representative images showing tube-liked structures within the 3 mm (B5) up to 9 mm (B7) regions. B1: The first 3 mm strip of Matrigel from general suture for the control, B2: Second 3 mm strip of Matrigel from general suture, B3: third 3 mm strip of Matrigel from general suture, B4: Fourth 3 mm strip of Matrigel from general suture, B5: The first 3 mm strip of Matrigel from NaBu Ultrabraid suture, B6: Second 3 mm strip of Matrigel from SB Ultrabraid suture, B7: Third 3 mm strip of Matrigel from SB Ultrabraid suture, B8: Fourth 3 mm strip of Matrigel from NaBu Ultrabraid suture. (**C**) shows relative release of NaBu from the suture into carefully processed 3 mm strips of matrigel alone (blue) or in the presence of BAEC (orange) moving away from the suture after 24 h. All experiment were repeated at least twice and representative examples are shown.

Straight lengths of the suture were placed in 6-well plates within Matrigel^TM^ gel and all other parameters were the same as per the standard Matrigel^TM^ gel assay. Normal suture did not stimulate tube formation, however, the UltraBraid + NaBu suture induced notable tube formation within the first 3–6 mm around the suture, and a lesser stimulatory effect from 9–12 mm (Fig. 4). In order to define if butyrate released from the suture was responsible for this, we measured butyrate levels in each 3 mm strip using HPLC and the data showed that significant quantities of butyrate were found after 24 h within the first 6 mm of Matrigel^TM^ gel (approximately 16–12 and 8–6 μg/ml in the first and second 0033 mm slices respectively, over a period of 24 h; without and in the presence of BAEC respectively) (Fig. 4B).

### Butyrate induced angiogenic cell signalling in BAECs through VEGFR2 and PKCβ1

**Figure 5:**
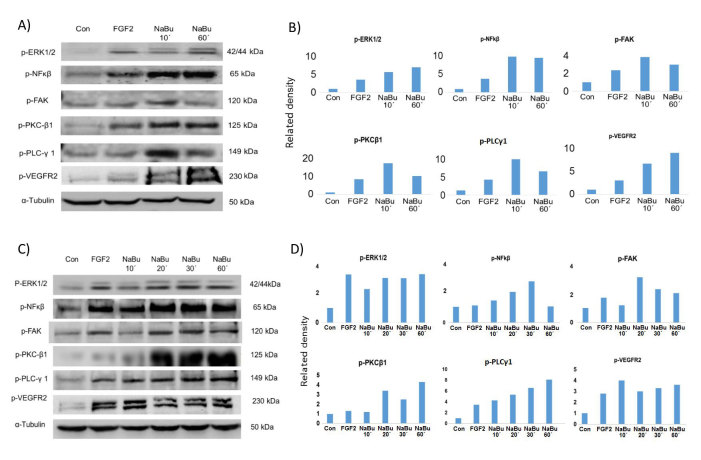
Western blotting shows that phosphorylated proteins, identified from the Kinexus phospho-protein screen were up-regulated by NaBu. (**A**) Western blot confirms the phosphorylation of PKCβ1, FAK, ERK1/2, p-VEGFR2 and NFkβ from the samples used in the Kinexus phospho-protein assay after 10/60 minutes exposure to NaBu 1 μg/ml. The bar charts (**B**) shows the relative intensity of the protein compared with control (1.0) expressions. (**C**) indicates the phosphorylated proteins in additional time-pointed (10–60 minute) sodium butyrate treated BAEC. All experiment were repeated at least twice and representative examples are shown.

A targeted Kinexus phospho-protein microarray employing stringently validated antibodies to over 500 target proteins and duplicate fields, was performed where BAEC were exposed to sodium butyrate (1 μg/ml/8 minutes and 1 h) and compared with control cells. Using their software packages where normalised signals use 100%CFC corresponding to a 2-fold increase, increases in phosphorylation of VEGFR2, PKCβ1, FAK, ERK1/2, NFκβ were seen within 8 minutes (Fig. 5A; Kinexus raw data included as Supplementary data-1). Western blotting confirmed these results showing strong up-regulation of phosphorylated proteins in treated cell lysates ranging from 10–60 minutes exposure (Fig. 5C). Figure 5B,D show fold differences in protein expression measured by image J compared to control expression (1.0).

### Inhibitor cell signalling blocking studies

**Figure 6:**
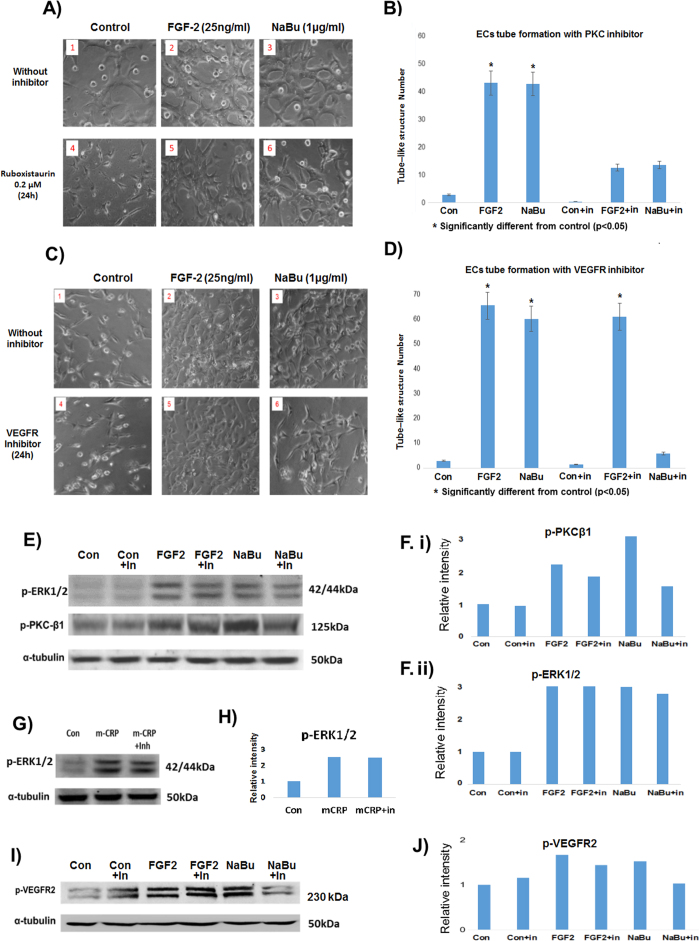
NaBu induced tube formation was inhibited in the presence of inhibitors of PKCβ and VEGFR2. (**A**) Shows the NaBu signalling pathway was blocked by the specific PKC inhibitor (Ruboxistaurin; 0.2 μM), and (**C**) shows the NaBu effect was inhibited by VEGFR2 blocking antibody (ABS553). Note that FGF-2 also stimulated phosphorylation of the VEGFR2, however this was not notably inhibited after pre-incubation with blocking antibody (see Discussion for explanation). (**E**,**F**) Shows strong inhibition of PKCβ1 phosphorylation induced by NaBu and a much weaker inhibition of FGF-2-induced PKC. G and H show the specificity of the PKCβ1 inhibitor as it did not inhibit mCRP-induced phosphorylation of p-ERK1/2 (a) down-stream signalling intermediate of the mitogenic pathway). All experiment were repeated at least twice and representative examples are shown.

Inhibitors of PKCβ1 (ruboxistaurin, optimised to 0.2 μM in our unpublished pilot studies) and/or VEGFR2 (blocking antibody; Millipore; ABS553) blocked protein/receptor phosphorylation respectively (Fig. 6E,G,I). Both VEGFR2 inhibition and PKCβ1 blocking, inhibited subsequent tube-like-structure formation in matrigel (Fig. 6A,C). As a positive control, FGF-2 tube-like structure formation and signalling were not affected when using the VEGFR inhibitor, demonstrating a level of specificity of the inhibitors. In addition, mitogenic signalling of another positive control protein (mCRP), through p-ERK 1/2 was also not affected by the inhibitor demonstrating the selectivity of our inhibitor at the concentrations used (Fig. 6G). Figure 6F,H,J show fold differences in protein expression measured by image J compared to control expression (1.0).

### *In vivo* subcutaneous implants of sodium butyrate elicited a pro-angiogenic phenotype

**Figure 7:**
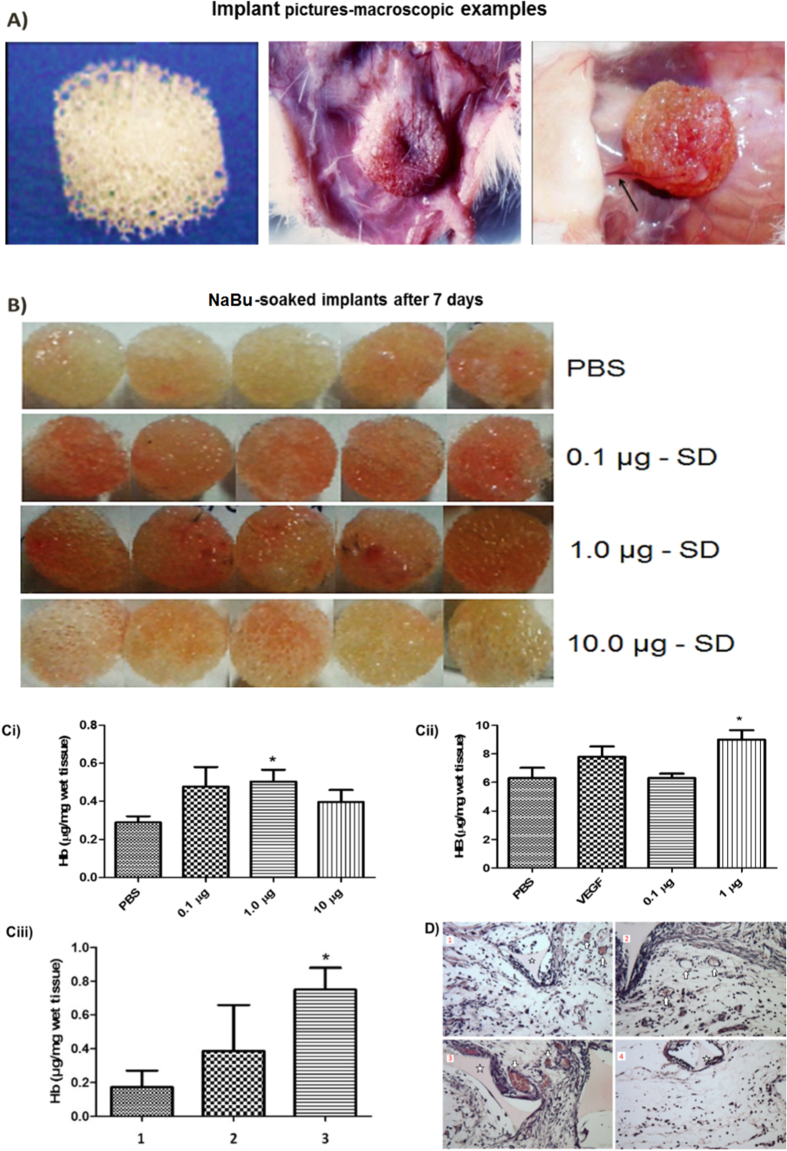
Haemoglobin analysis after 7 days sponge implantation with NaBu. The macroscopic examination of the sponge matrix showed that sodium butyrate induced vascularization and blood vessels within the implants, as seen in the representative images of the sponges delivered subcutaneously (**D**). This alteration was further confirmed in parts by increase in the haemoglobin content (vascular index) in the sponge implants treated with 0.5–1.0 μg of sodium butyrate and vessels number showed by histological sections treated with 0.1 and 1.0 μg. (**A**) Implant pictures-macroscopic examples. (**B**) NaBu -soaked implants after 7 days. (D1): Sponge only, (D2): Sponge + general suture, (D3): Sponge + NaBu Ultrabraid suture. (D4): no suture and no sponge. All experiment were repeated at least twice and representative examples are shown.

Using the sponge implant model (mouse), we showed that sodium butyrate soaked sponges (0.1–1.0 μg/ml) demonstrated a notable increase in Hb influx (Fig. 7A,B,D), and the number of new microvessels within the matrix measured after 7 days (there were no vessels in the sponge vicinity at the start of the experiment) (Fig. 7C), although levels of VEGF were not found to be elevated (data not included).

## Discussion

It is generally reported that NaBu is an inhibitor of histone deacetylases (HDACs)^35^,^36^,^37^, and this inhibition has been utilised in relation to cancer; however, from a vascular perspective at high concentrations (>500 μg/ml) butyrate inhibited VEGF production by glioblastoma cells^28^, The most recent paper in this area by Kim and Chuang^38^ found a similar result to ours in that NaBu protected against ischaemia-induced injury in a rat model-in part at least through up-regulation of VEGF protein. From our phospho-screening results, at the pro-angiogenic concentrations tested, only PKC could be linked an HDACs signalling network through GPCR/TRPC and we suggest here that at these very low concentrations (0.1–1.0 μg/ml), HDAC pathways were not significantly modulated.

Several studies have shown and defined that NaBu acts classically as a potent anti-angiogenic agent in tumour angiogenesis models *in vivo* and *in vitro*^13^,^27^,^39^. However, previously published evidence by our consortium established a potentially beneficial effect of UltraBraid+NaBu in a rabbit -bone healing model with evidence of positive tissue remodelling and local angiogenesis^40^. Therefore, here, we evaluated the possible pro-angiogenic effects of NaBu *in vitro* and in an inflammatory angiogenesis model induced by sponge implants in mice.

Of primary importance initially, was to determine the relative release rate of butyrate from the UltraBrade+NaBu suture to indicate the likely micro-environmental concentrations cells and tissues would be exposed to *in vivo*. We showed that sodium butyrate release from the suture into Matrigel^TM^ gel ECM was in the order of magnitude of 5–10 μg/ml over a 24 h period diffusing up to around 1cm or more from the suture-and being an order of magnitude greater than normal tissue concentrations of butyrate^15^. Taking into account the kinetics of NaBu metabolism, we estimated a micro-environmental dynamic expression of between 0.1–1.0 μg/ml over the first 24–72 h. Hence, we needed to identify what effects if any this level of release would have on EC/BAEC activation and angiogenesis.

Our *in vitro* angiogenesis studies revealed that sodium butyrate at concentrations of between 25 ng/ml and 5 μg/ml were strongly angiogenic to vascular endothelial cells, stimulating migration, tube-like-structure formation and invasion from one layer of Matrigel^TM^ gel to another. Although we have not detected in the literature previously published evidence of butyrate promoting angiogenesis *in vitro*, recent evidence has suggested a beneficial role on tissue remodelling *in vivo*. Combination treatment of dimethyloxalglycine and butyrate improved bone regeneration and angiogenesis concomitant with increasing expression levels of VEGF in a rat model^41^ whilst Chen Y. *et al*.^42^ showed that the presence of butyrate increased angiogenesis in diabetic rat hearts, also inhibiting cardiac hypertrophy. It would appear the micro-environmental concentration of sodium butyrate within the tissue might determine its biological activity as we also showed that concentrations above 10 μg/ml were not able any more to stimulate angiogenesis *in vitro*. As an anti-cancer agent, *in vitro* studies have suggested inhibition of growth at concentrations of 1 μg/ml and above-e.g. inhibiting neuroblastoma cell growth, inducing apoptosis and reducing VEGF production by the tumour cells^43^. Hence the effects on cells may be cell-specific. This data strongly support a positive role for the Ultrabraid+NaBu suture in supporting angiogenesis and positive tissue remodelling in tendon-bone healing.

We investigation of the signalling mechanism through which sodium butyrate induced angiogenesis by firstly conducting a targetted Kinexus phospho-protein microarray after 10 minutes and 1 h exposure to 1 μg/ml. The results indicated increased expression of several proteins linked to angiogenesis, most interestingly, VEGFR2, PKCβ1, PLC-ɣ1 and FAK. The strong up-regulation of these phosphorylated proteins was confirmed by Western blotting. Angiogenesis stimulated through VEGFR2-PLC-PKC and ERK1/2^44^ and concomitantly VEGFR2-FAK^45^ are conventional/common signalling mechanisms. Here, we showed that blocking of the VEGFR2 directly with inhibiting antibodies, prevented sodium butyrate-induced angiogenesis and in addition, the PKCβ1 specific inhibitor-ruboxistaurin^46^ produced similar effects without affecting FGF-2 – induced signalling and angiogenesis. The most likely explanation for these results is that sodium butyrate was able to stimulate increased production of growth factors such as VEGF and this has been demonstrated recently by^38^, who showed that butyrate treatment of rats following middle cerebral artery occlusion, resulted in significant increase in VEGF expression in the ischaemic striatum. Our FGF-2 control samples also stimulated phosphorylation of the VEGFR2-as shown in our Western blotting studies, and this autocrine stimulation of VEGF has been reported several times previously within the literature^47^.

*In vivo*, we conducted a sponge implant study to test the impact of sodium butyrate. This model has been adopted as a model for the accurate quantification of angiogenic and fibrogenic responses as they occur during wound healing *in vivo*. The macroscopic examination of the sponge matrix showed that sodium butyrate induced vascularization and blood vessels within the implants, as seen in the representative images of the sponges delivered subcutaneously. This alteration was further confirmed in parts by increase in the haemoglobin content (vascular index) in the sponge implants treated with 0.5–1.0 μg of sodium butyrate and vessels number showed by histological sections treated with 0.1 and 1.0 μg. This data taken together providing *in vivo* evidence that low concentrations of NaBu induced angiogenesis in mice. Our data are consistent, also, with other *in vivo* studies demonstrated that NaBu was associated with enhanced angiogenesis in models of repair and healing of tendons/meniscus and in an *in vivo* model of local cerebral ischemia concomitant with upregulation of VEGF^38^,^40^,^48^,^49^.

In order to know what would be the promoter of the angiogenic process induced by NaBu in sponge implant model, we evaluated the levels of VEGF, which we hypothesise may be primarily responsible for eliciting these effects. It has been reported that VEGF levels occur to a greater extent and more consistently than other angiogenic factors in angiogenesis *in vivo* models and in our study the VEGF levels was significantly increased when treated with 10 μg sodium butyrate but not in lower concentrations. It is possible that higher VEGF levels in the least vascularized implants (10 μg sodium butyrate treatment) would be a compensatory mechanism to stimulate blood vessel formation in the environment of the implants. Furthermore, we suggest that other signalling mechanisms could be influencing the angiogenic process in implants treated with low doses of VEGF.

In this work, we wanted to provide a rationale for the observed improvement seen in tendon-bone healing with concomitant histological evidence of better vascular networking and improved tissue remodelling^48^. Using the butyrate-soaked suture in comparison with standard suture, we showed that the Ultrabraid + NaBu suture was able to induce substantial endothelial tube-like-structure formation in matrigel in the local micro-environment up to approximately 1cm distant from the edge of the suture. No angiogenic effect was seen with standard suture. In addition, quantification of the amount of sodium butyrate released showed that it was of the order of magnitude that might coincide with *in vivo* stimulation of tissue remodelling.

Overall, NaBu has been shown to stimulate angiogenesis at low doses in a vascular repair model *in vivo* and any detected pro-angiogenic changes demonstrates the potential of this compound to stimulate repair in a wound healing at a site where vasculature is low and healing is compromised.

## Additional Information

**How to cite this article**: Liu, D. *et al*. Low Concentration of Sodium Butyrate from Ultrabraid+NaBu suture, Promotes Angiogenesis and Tissue Remodelling in Tendon-bones Injury. *Sci. Rep.* **6**, 34649; doi: 10.1038/srep34649 (2016).

## Supplementary Material

Supplementary Information

## Figures and Tables

**Table 1:** Animal study design, tests performed and number of animals (replicates) per test.

NaBu total dose (μg)	Test and Replicate Number	
	Haemoglobin (Hb) N-acetyl-β-D-glucosaminidase (NAG) VEGF Analysis	Histology
0.1	8	5
0.5	8	5
1.0	8	5
5.0	8	5
10.0	8	5
50.0	8	5
100.0	8	5
0 (Control)	8	5
**Total animals**	**64**	**40**
